# Role of the ubiquitin proteasome system in Parkinson's disease

**DOI:** 10.1186/1471-2091-8-S1-S13

**Published:** 2007-11-22

**Authors:** Kah-Leong Lim, Jeanne MM Tan

**Affiliations:** 1 Neurodegeneration Research Laboratory, National Neuroscience Institute, Singapore; 2 Parkinson's Disease and Movement Disorders Center, National Neuroscience Institute, Singapore; 3 Department of Biological Sciences, National University of Singapore, Singapore

## Abstract

Parkinson's disease (PD) is the most common neurodegenerative movement disorder. Although a subject of intense research, the etiology of PD remains poorly understood. Recently, several lines of evidence have implicated an intimate link between aberrations in the ubiquitin proteasome system (UPS) and PD pathogenesis. Derangements of the UPS, which normally functions as a type of protein degradation machinery, lead to alterations in protein homeostasis that could conceivably promote the toxic accumulation of proteins detrimental to neuronal survival. Not surprisingly, various cellular and animal models of PD that are based on direct disruption of UPS function reproduce the most prominent features of PD. Although persuasive, new developments in the past few years have in fact raised serious questions about the link between the UPS and PD. Here I review current thoughts and controversies about their relationship and discuss whether strategies aimed at mitigating UPS dysfunction could represent rational ways to intervene in the disease.

**Publication history: **Republished from Current BioData's Targeted Proteins database (TPdb; ).

## Parkinson's disease

Parkinson's disease (PD) is the most common neurodegenerative movement disorder, affecting millions of elderly individuals worldwide [1,2]. Clinically, the disease is characterized by major motoric difficulties that include bradykinesia, postural instability, rigidity and tremor. Although neurodegeneration in PD involves multiple areas of the brain such as the dorsal motor nucleus of the vagus, locus ceruleus (LC) and olfactory nuclei [3], the principal neuropathology that gives rise to the array of motoric deficits seen in PD patients is the loss of dopaminergic neurons in the substantia nigra pars compacta (SNpc), which results in a severe depletion of striatal dopamine (DA) [4]. Often, the neuronal degeneration is accompanied by eosinophilic intracytoplasmic inclusions known as Lewy bodies (LBs) that are present in surviving neurons of the SN as well as in other affected brain areas [5]. However, the LB is not an obligate marker of the disease as some familial PD cases are apparently devoid of its presence [6-9].

Although a subject of intense research, the etiology of PD remains poorly understood and no treatment exists to prevent, cure or retard the progression of this disabling disease. Current favored hypotheses for sporadic PD include combinations of the aging process, genetic propensity and environmental exposures leading to oxidative stress, mitochondrial dysfunction, microglial activation and excitotoxicity [2,10]. More recently, evidence from several lines of study strongly implicate a key role for the ubiquitin proteasome system (UPS) in PD pathogenesis [11-14].

## UPS

**Figure 1:**
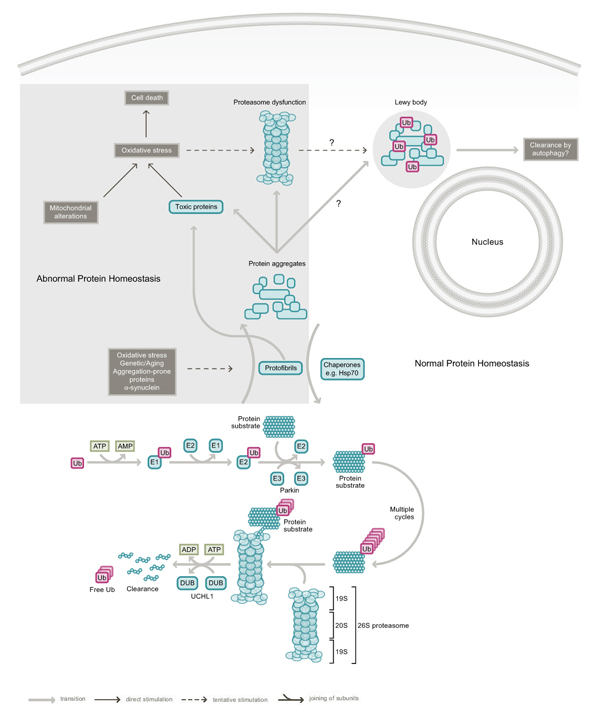
**The ubiquitin proteasome system (UPS) and Parkinson's disease (PD)**. Under normal conditions, proteins destined for proteasomal degradation are tagged with a chain of ubiquitin (Ub) proteins via multiple rounds of a linear reaction catalyzed by ubiquitin activating (E1), conjugating (E2) and ligating enzymes (E3). An example of an E3 is parkin. Ubiquitylation reactions are reversed by the action of deubiquitylating enzymes (DUBs), of which UCHL1 is a member. Energy in the form of ATP is required to drive the UPS machinery. Age-related changes, exogenous stress, mitochondrial alterations and PD-linked genetic mutations in parkin, UCHL1 and α-synuclein could promote disruption of the UPS and conceivably result in the accumulation of protein aggregates or abnormal protein intermediates that could be directly detrimental to neuronal survival. Lewy bodies are thought to form as a result of an attempt by the cell to sequester these abnormal proteins. The enhancement of protein re-folding by chaperones such as Hsp70 and the clearance of protein aggregates via the stimulation of autophagy could help mitigate UPS dysfunction. Such strategies may offer innovative approaches in the treatment of PD.

The UPS is an intracellular protein degradation system that is responsible for the majority of protein turnover within the cell [15] (Figure 1). In this system, proteins destined for degradation are covalently tagged with ubiquitin, a 76 amino acid residue protein, through the formation of an iso-peptide bond between the ε-amino group of a lysine residue of the substrate and the C-terminal carboxylate (G76) of ubiquitin. This ligation reaction is elaborate and requires the sequential actions of ubiquitin activating (E1), conjugating (E2) and ligating (E3) enzymes [16] (Figure 1). Usually, the multienzyme-mediated ubiquitylation process is repeated many times to allow the formation of a polyubiquitin chain on the substrate. Ubiquitin molecules in the chain are linked together via iso-peptidic bonds between the terminal G76 residue of each ubiquitin unit and a specific lysine (K) residue (most commonly K48) of the previous ubiquitin [17]. The G76–K48 polyubiquitylated substrate is then targeted for degradation by the 26S proteasome, a large protease complex consisting of a barrel-shaped 20S proteolytic core in association with two 19S (PA700) regulatory caps, one on each side of the barrel's openings (Figure 1). The 20S catalytic core is characterized by three distinct proteolytic activities: chymotrypsin-like, trypsin-like and peptidyl glutamylpeptide hydrolytic, which can be measured *in vitro* by means of a fluorimeter using Suc-Leu-Leu-Val-Try-AMC, Boc-Leu-Arg-Arg-AMC and Z-Leu-Leu-Glu-AMC, respectively, as fluorogenic peptide substrates [18]. The components of the 19S cap play vital roles in the initial steps of substrate proteolysis, including the recognition, unfolding and translocation of substrate proteins into the lumen of the proteolytic core [19-21]. Individual ubiquitin monomers are regenerated in the process by the actions of deubiquitylating enzymes (DUBs) (Figure 1). It is important to note that the ubiquitin molecule contains a total of seven lysine residues (at positions 6, 11, 27, 29, 33, 48 and 63) and that polyubiquitin chain assembly via alternative lysine linkages has also been described [22]. Notably, K63-linked polyubiquitylation of proteins is not typically associated with proteasomal degradation [23-27]. It is also noteworthy that an enormous number of cellular proteins (>1000), comprising various E1s, E2s, E3s and other related members, are involved in the UPS [28]. Indeed, protein ubiquitylation rivals protein phosphorylation as a major post-translational event in the cell. Conceivably, therefore, derangements of UPS function could lead to dire cellular consequences, particularly in post-mitotic cells.

## UPS and PD

Evidence implicating a direct role for the UPS in PD came from the association of genetic mutations in the *parkin* (*PRKN2*) gene with familial parkinsonism about a decade ago [13], and the subsequent demonstration by three independent groups that parkin functions as a ubiquitin ligase associated with proteasomal degradation [29-31]. Importantly, using *in vitro* assays to measure the ubiquitylation activity of parkin immunoprecipitated from transfected cells, these pioneer investigators found that disease-causing mutations in parkin compromise its normal role as an E3 enzyme [29-31]. This property of parkin thus directly links UPS aberrations to dopaminergic neuronal survival, the association of which is further supported by the discovery of a missense mutation (I93M) in UCHL1, a DUB, in a pair of German siblings with inherited PD [14]. Notably, the I93M UCHL1 mutant has markedly reduced ubiquitin hydrolase activity *in vitro*[14], suggesting that impaired polyubiquitin hydrolysis could also contribute to dopaminergic neuronal death. Another PD-linked gene product, α-synuclein, is a major component of LBs [32]. Although α-synuclein is not a member of the UPS, overexpression of wild-type or mutant α-synuclein in cultured cells and in the brains of transgenic mice is known to inhibit proteasome function (as determined by *in vitro* 20S proteasome assay), with disease-relevant mutants eliciting stronger effects [33-35]. Notably, triplication of the α-synuclein gene is also causative of PD [36].

Consistent with an implicated role for the UPS in familial parkinsonism, McNaught and colleagues (Mount Sinai School of Medicine) observed a significant reduction in the levels of PA700 expression in the SNpc of post-mortem sporadic PD brains relative to control brains [11] (PA700 levels are otherwise elevated in other regions of the PD brain such as the frontal cortex and striatum [11,37]). Selective decrease in the level of proteasomal core subunits within the SN was also noted [11]. Further, *in vitro* assay of SN extracts from PD brains revealed a marked decrease in the activity of the 20S proteasome [11]. Corroborating evidence came from a parallel report by Spillantini and co-workers (Cambridge Centre for Brain Repair) who demonstrated a 55% reduction in the proteasomal chymotrypsin-like activity in the SN, but not in the cingulate, frontal and occipital cortices, of PD brains [38]. Together, these results suggest structural and functional impairments of the UPS in sporadic PD. Although the precise basis of UPS dysfunction in sporadic PD awaits further clarifications, it likely involves age-related changes in the brain resulting in increased oxidative stress and energy depletion, as well as exposures to environmental toxins [39,40]. Supporting this, the herbicide maneb and a range of pesticides linked to sporadic PD, including rotenone, have been shown to produce proteasome inhibition *in vitro*[41,42]. Similarly, we recorded a marked decrease in proteasome activity in extracts derived from rotenone- and paraquat-treated cells relative to untreated controls [43]. Further, Betarbet *et al.* (Emory University) found that proteasome function is selectively decreased in the ventral midbrain region in rotenone-induced rats [42], while a similar study by another group (University of Pisa) showed that continuous infusion of the parkinsonian neurotoxin, MPTP, in mice induces proteasome impairments in their brains [44]. Taken together, these results suggest a co-operative relationship between environmental exposures and proteasome dysfunction in promoting neurodegeneration in sporadic PD.

How does failure of the UPS lead to dopaminergic neurodegeneration? Conceivably, aberrant protein homeostasis could result in the toxic accumulation of intracellular proteins detrimental to neuronal survival. Supporting this, the accumulation of misfolded and aggregated α-synuclein is thought to be the primary pathogenic event in familial PD linked to mutations or multiplication of the α-synuclein gene [45]. Notably, aggregated α-synuclein selectively interacts with the 19S cap and concomitantly inhibits the function of the 26S proteasome [46], thereby perpetuating a vicious cycle. Similarly, in parkin-linked parkinsonism, the accumulation of certain parkin substrates could interfere with cellular functions and induce neurotoxicity [45]. Indeed, numerous reported substrates of parkin, including CDCrel (cell division control-related protein) 1 and 2a [47], Pael-R (parkin-associated endothelin-like receptor) [48], cyclin E [49], p38 (JTV-1/AIMP2) [50] and FBP1 (far upstream sequence element-binding protein 1) [51], accumulate in the brains of PD patients carrying parkin mutations. Further, both p38 and FBP1 also accumulate in the brains of parkin-deficient mice [50,51], and overexpression of p38 in cell culture or SN of mice leads to marked neurotoxicity [50]. Taken together, these results suggest that parkin-mediated ubiquitylation of its substrates promotes their turnover via the UPS and offer a simple hypothesis that toxic substrate accumulation in the absence of functional parkin triggers neurodegeneration. However, because none of these parkin substrates are exclusively expressed in dopaminergic neurons, it remains puzzling as to why dopaminergic neurons are selectively vulnerable to parkin deficiency. Nonetheless, the importance of parkin function to neuronal survival is currently widely accepted. This is exemplified by its broad neuroprotective capacity, which includes conferring protection against manganese-induced cell death [52], α-synuclein toxicity [53], Pael-R [54] and p38/JTv-1 accumulation [50], kainate-induced excitotoxicity [49], DA-mediated toxicity [55], and importantly, proteasome dysfunction [43,56]. Accordingly, the loss of parkin function following its mutation is expected to compromise neuronal survival. Interestingly, a recent ubiquitylation assay conducted by Tanaka (Tokyo Metropolitan Institute of Medical Science) and Corti (INSERM) laboratories using purified recombinant parkin demonstrated that the majority of disease-associated parkin missense mutations do not compromise its enzymatic activity *in vitro*[57,58]. Conversely, several other groups, including ours, found that parkin mutations often alter the protein's solubility and concomitantly promote its aggregation within the cell [59-62]. Further, we and others also found that normal brain aging, as well as a wide variety of PD-linked stressors, including DA, induce similar alterations in parkin [43,63,64] or otherwise inactivate the enzyme [65,66], thereby suggesting a mechanism for parkin dysfunction in the pathogenesis of idiopathic PD. Loss of soluble, functional parkin resulting from normal aging or stress-induced modifications could therefore be as relevant to sporadic PD as parkin dysfunction triggered by disease-associated mutations is to parkin-related parkinsonism.

## Disease models, knockouts and assays

Using fetal rat ventral mesencephalic cultures as a model, several groups have demonstrated that lactacystin-induced proteasome dysfunction results in a dose-dependent and preferential degeneration of dopaminergic neurons, accompanied by the formation of inclusion bodies that stain positively for ubiquitin and α-synuclein [67,68]. Interestingly, inactivation of ubiquitin hydrolases with ubiquitin aldehyde produces similar effects in these primary cultures [67]. However, these studies were performed using acute doses (5–10 µM for 24–48 hours) of pharmacological inhibitors. To better reflect the events occurring in susceptible neurons during PD pathogenesis, Keller and co-workers (University of Kentucky) subjected SH-SY5Y neuroblastoma cells to low levels of proteasome inhibition (100 nM MG115) for an extended period lasting several weeks. They observed in these cells elevated levels of protein oxidation and protein aggregates, as well as altered mitochondrial homeostasis [69,70]. Neural cells exposed to chronic proteasome inhibition thus reproduce the key features of sporadic PD faithfully [69,70], thereby supporting the involvement of UPS aberrations in PD pathogenesis. Consistent with this, two groups observed retrograde dopaminergic neurodegeneration in rodent brains following intrastriatal administration of lactacystin [71,72]. In further pursuit of this hypothesis, McNaught and colleagues subjected adult Sprague-Dawley rats to six regularly-spaced subcutaneous injections of either the naturally occurring proteasome inhibitor epoxomicin (1.5 mg/kg) or the synthetic proteasome inhibitor PSI (3 or 6 mg/kg) over a period of two weeks [73]. Remarkably, after a latency of two weeks, both inhibitors induced progressive motor dysfunction characterized by bradykinesia, rigidity and tremor in treated rats that could be alleviated by L-DOPA administration [73]. Consistent with the loss of dopaminergic nerve terminals in the striatum, ^11^C-CFT-based positron emission tomography imaging revealed a progressive reduction of the DA transporter ligand in these rats [73]. The nigral pathology, as well as extra-nigral degeneration in brain regions such as the LC, dorsal motor nucleus of the vagus and nucleus basalis of Meynert, was confirmed at post mortem [73]. Whereas a reduction in proteasome activity, as measured *in vitro*, appears to accompany the degeneration, elevated proteasome activity was observed in areas that remained intact, suggesting a compensatory mechanism [73]. Notably, inclusions that stained positively for ubiquitin and α-synuclein formed within neurons that survived the degeneration process in affected areas [73]. This rat model of PD, based on systemic administration of proteasome inhibitors, thus recapitulates the key clinical and pathological hallmarks of PD faithfully and provides solid support for the role of UPS disruption in PD pathogenesis. Unfortunately, the model is currently controversial as several laboratories have recently reported failures in their attempts to replicate the results despite diligently adhering to McNaught's original protocol [74-76]. These conflicting findings include the absence of nigral pathology, motor impairment and neuronal inclusions [74-76]. However, some other groups did manage to reproduce the nigral pathology [77,78]. Though the reason for this variability is currently unclear, it could be due to variations in the source and hence quality of the inhibitors, differences in methodologies or exogenous factors present in feeds or in the environment [79,80]. Hopefully, these issues will soon be resolved as the novel model of PD generated by McNaught and colleagues is arguably one that reflects the disease most accurately and as such has important implications for the development of PD therapeutics.

Concurrent to the proteasome inhibition model, several investigators have also generated genetic models of PD to probe the link between the disruption of key UPS players and PD pathogenesis. Two groups, led by Shen (Harvard Medical School) and Rooney (Aventis Pharma) respectively, first reported mouse models that are ablated of orthologous *parkin*[81,82]. However, none of these models exhibit parkinsonian phenotypes. Indeed, with the exception of cell loss in the LC in a subsequent model generated by the Dawson laboratory (Johns Hopkins School of Medicine) [83], parkin null mice have generally failed to exhibit apparent signs of parkinsonism [81,82,84]. Further, the majority of previously identified parkin substrates (except p38 and FBP) do not show any detectable accumulation in these mice [50,51,81,82,84]. Similarly, inactivation of UCHL1 in mice leads not to dopaminergic neuronal death but to gracile axonal dystrophy syndrome, a degenerative disease associated with sensory and motor ataxia [85]. However, *Drosophila* parkin null mutants, originally developed by Greene *et al.* (University of Washington) [86], exhibit selective dopaminergic neurodegeneration in the adult brains and concomitant locomotion defects [87,88], a phenotype that appears to mirror the human condition. Although the anatomy of the *Drosophila* brain differs from that of the vertebrate brain, many aspects of nervous system development and function are conserved between flies and humans. The parkin fly model could thus offer opportunities for the discovery and testing of experimental PD therapeutics, especially those aimed at restoring parkin function or compensating for parkin dysfunction.

## Disease targets and ligands

Since disruption of the UPS appears to play a key role in PD pathogenesis, therapies aimed directly or indirectly at mitigating UPS dysfunction could potentially offer respite for individuals afflicted with PD. Some strategies aimed at alleviating UPS dysfunction are discussed below.

(i) *Managing protein misfolding and aggregation* – The accumulation and subsequent aggregation of proteins as a result of proteasome inhibition is a likely contributor to pathogenicity. Conceivably, strategies aimed at reducing proteasomal load or stimulating proteolysis could promote beneficial outcomes for the PD patient. This could involve decreasing the burden of pathogenic proteins through RNA silencing [89]. Alternatively, gene-based approaches that enhance the expression levels of chaperones to facilitate protein re-folding could also be effective [90-92]. For example, co-expression of hsp70/Hsp70 with α-synuclein reduces α-synuclein aggregation and concomitantly attenuates α-synuclein-mediated dopaminergic neuronal death in both flies and mice [90,92]. Further, Hsp70 gene transfer by adeno-associated virus vector inhibits MPTP-induced nigrostriatal degeneration in mice [91], presumably by reducing the levels of misfolded proteins induced by the neurotoxin. Not surprisingly, pharmacological promotion of Hsp70 expression by Geldanamycin, a commercially available drug whose analogs are currently entering/undergoing Phase II clinical trials, also mitigates neurotoxicity in several models of PD [93,94]. Aside from these, compounds that directly stimulate UPS activity are also expected to reduce protein aggregation and pathology. Dexamethasone, an anti-inflammatory drug commercially marketed under brand names such as Decadron and Solurex, is known to increase the expression levels of various UPS components including subunits of the proteasomal core and PA700 activator [95]. These combined effects could explain dexamethasone's protection against dopaminergic neurodegeneration in MPTP-treated mice [96]. Potentially, several other UPS modulators currently being tested for varied disease conditions could also be relevant to PD (see UPS Drugs and Biologicals table).

(ii) *Autophagy induction* - Autophagy is a major cellular process by which cytoplasmic components, including organelles, are catabolized [97,98]. Although autophagy represents a distinct degradation system from the UPS, recent evidence suggests a complementary relationship between these two systems in the maintenance of protein homeostasis [99]. Further, two recent independent reports provide compelling evidence regarding the importance of autophagy in neurodegeneration [100,101]. Of particular relevance to PD, both the UPS and autophagy are apparently involved in α-synuclein clearance [102]. Harnessing this pathway could thus offer innovative approaches in the treatment of PD. The mammalian target of rapamycin (mTOR) of the phosphatidylinositol 3-kinase pathway, which normally functions to shut down autophagy, represents a good target to modulate the lysosome-mediated degradation system [97,98]. Rapamycin-mediated inhibition of mTOR activates autophagy and increases the clearance of aggregate-prone proteins, including α-synuclein, and concomitantly reduces their neurotoxicity [103]. Similarly, activation of autophagy via mTOR-independent pathways through the actions of lithium (which inhibit inositol monophosphatase thereby leading to the activation of autophagy) or trehalose (mechanism of activation unknown), promotes beneficial outcomes in cell culture models of protein aggregation [104,105].

(iii) *Parkin gene therapy* – Given that parkin apparently functions as a broad-spectrum neuroprotectant, it is conceivable that *parkin* gene delivery could offer a novel avenue of PD therapy. Indeed, virus-mediated delivery of parkin prevents dopaminergic neurodegeneration in rats either overexpressing α-synuclein [106,107] or treated with 6-Hyroxydopamine (6-OHDA, a catecholaminergic neurotoxin that triggers cell death through the generation of oxidative stress [108]), as well as in mice treated with MPTP [109]. Concurring with these, a recent study further demonstrated the protective function of parkin delivery in a non-human primate model of α-synuclein overexpression [110]. Similarly, enhanced expression of parkin in *Drosophila* counteracts PD-like symptoms promoted by the overexpression of α-synuclein [111] or Pael-R [54]. Interestingly, a recent screen for modifiers of parkin dysfunction in flies identified glutathione S-transferase S1 (GstS1) [88]. Overexpression of GstS1 prevents dopaminergic neurodegeneration in parkin mutant flies [88], suggesting that the induction of GstS1 expression could be useful in the treatment of PD.

Although the above strategies aimed at mitigating UPS function could represent promising therapeutic approaches for the PD patient, it is important to note that sporadic PD is likely multifactorial and that the UPS could just be one of several players responsible for its pathogenesis. Given this, PD could perhaps be cured not through the administration of a singular therapy, but through a combination of therapies targeted at major problem centers in the pathogenic cascade, with the UPS possibly being one of the focal points.

## Next frontiers

Despite what appears to be compelling evidence implicating an association between the UPS and PD, certain gaps and controversies surrounding their relationship need to be clarified before we can move forward with UPS-based therapeutics. One of these relates to the validity of parkin and UCHL1 mutations in supporting a role for the UPS in PD. Although the association of parkin and UCHL1 mutations with familial parkinsonism supports a link between the UPS and PD, it is apparent that the involvement of the UCHL1 I93M mutation in PD pathogenesis has become contentious in recent years as its occurrence to date is restricted to the pair of German siblings [112]. Furthermore, novel roles for parkin-mediated ubiquitylation that are uncoupled from the proteasome are progressively being elucidated. Emerging evidence suggests that parkin is a multifaceted ubiquitin ligase capable of mediating alternative modes of ubiquitylation that are not typically associated with proteolysis [57,58,113,114]. These non-proteolytic forms of parkin-mediated ubiquitylation, which include K63-linked polyubiquitylation and monoubiquitylation, have been linked to diverse cellular processes such as DNA repair, IkBa kinase activation, endocytosis and translational regulation [23-27], thereby suggesting that parkin-mediated ubiquitylation could play regulatory roles. Supporting this, Fallon *et al.* (Montreal Neurological Institute) recently identified Eps15, an adaptor protein that is involved in epidermal growth factor receptor endocytosis and trafficking, as a substrate of parkin-mediated monoubiquitylation [115]. Similarly, Hampe *et al.* (INSERM) demonstrated that parkin mediates multi-monoubiquitylation of p38 *in vitro*[57], an observation that apparently contradicts the finding by the Dawson laboratory that p38 accumulates in the brains of parkin-deficient mice and parkin-related PD patients [50]. The reason for this discrepancy remains unclear. Conversely, we have shown that parkin normally mediates K63-linked polyubiquitylation of the α-synuclein-interacting protein, synphilin-1, which promotes the stability of the protein and concomitantly its sequestration into LB-like inclusions [113]. Our result suggests the interesting possibility that parkin-mediated K63-linked ubiquitylation could contribute to LB biogenesis and at the same time provide an explanation to the general absence of LBs in parkin-related parkinsonism [6-8]. Depending on the mode of ubiquitylation, parkin thus appears to have the capacity to promote either degradation or stability of its substrates. This is seemingly paradoxical, but is consistent with parkin's multidimensional roles in the cell [116] and recent observations by several groups that previously identified parkin substrates (except p38 and FBP1) did not accumulate in the brains of parkin-deficient mice [50,81,82,84]. Importantly, it cautions against fixation on the traditional view that substrates of parkin must exhibit an accelerated, proteasome-dependent turnover in the presence of the enzyme. At present, it would appear that disruption of proteasome-independent events following parkin mutations could also account for the susceptibility of neurons to degeneration in patients with parkin-related parkinsonism. Clearly, considerably more basic work needs to be done to clarify the multifunctional roles of parkin, and its interactions with the UPS, be it direct or indirect. The concurrent elucidation of the determinants governing the choice of parkin-mediated ubiquitylation would be illuminating. Because antibodies that specifically recognize different ubiquitin chain topologies are currently unavailable, I envisage that mass spectrometry-based proteomics techniques would be useful here.

Another urgent endeavour is to resolve the conflicting findings between the proteasome inhibition models generated by McNaught and by others, since reproduction of the original model could provide unequivocal support for a direct role of UPS disruption in PD pathogenesis. Interestingly, Landau *et al.* (McGill University) recently repeated McNaught's experiment in mice and found that the ethanol vehicle produces marked dopaminergic neurotoxicity and significantly eclipses the effects of the synthetic proteasome inhibitor PSI in the dopaminergic system [117]. Notably, ethanol is known to elicit proteasome inhibition and oxidative stress in the liver [118]. The ethanol vehicle for PSI thus appears to be a confounding problem for this model. Meanwhile, two prominent PD researchers have suggested the generation of additional series of animals by the original laboratory and sending them to other laboratories for blinded assessment [79]. This will certainly help to validate the model.

Finally, the relationship between LB inclusions and UPS impairment remains controversial. While the presence of α-synuclein-enriched LBs is compatible with proteasome dysfunction, a recent study from Spillantini's laboratory found no generalized impairment of the proteasome in brain regions with LB pathology [38]. Obviously, a better understanding of how misfolded proteins are targeted for degradation or inclusion formation would be useful. It is possible, as we and others have recently speculated, that inclusion formation could represent attempts by the cell to divert misfolded proteins away from an overloaded proteasome (thus prolonging its survival), especially in times of chronic stress [119,120]. I anticipate that live cell imaging techniques coupled with a reliable *in vivo* proteasome reporter assay would help to address some of the questions regarding the life cycle of an inclusion body and its relationship with the UPS.

## Abbreviations

DA, dopamine; GstS1, glutathione S-transferase S1; PD, Parkinson's disease; LC, locus ceruleus; SNpc, substantia nigra pars compacta; UPS, ubiquitin proteasome system.

## Competing interests

The authors declare that they have no competing interests.

## Publication history

Republished from Current BioData's Targeted Proteins database (TPdb; ).
